# Polypeptide *N*-acetylgalactosaminyltransferase-15 regulates adipogenesis in human SGBS cells

**DOI:** 10.1038/s41598-024-70930-5

**Published:** 2024-08-29

**Authors:** Asuka Takahashi, Ryo Koike, Shota Watanabe, Kyoko Kuribayashi, Martin Wabitsch, Masahiko Miyamoto, Akihiko Komuro, Mineaki Seki, Masayuki Nashimoto, Akiko Shimizu-Ibuka, Kikuji Yamashita, Takeo Iwata

**Affiliations:** 1 Department of Functional Morphology, Graduate School of Pharmaceutical Sciences, Niigata University of Pharmacy and Medical and Life Sciences, Niigata, 956-8603 Japan; 2 Department of Functional Morphology, Faculty of Pharmaceutical Sciences, Niigata University of Pharmacy and Medical and Life Sciences, 265-1 Higashijima, Akiha-ku, Niigata, 956-8603 Japan; 3 Department of Oral and Maxillofacial Surgery, Ehime University Graduate School of Medicine, Tōon, 791-0295 Japan; 4 Division of Pediatric Endocrinology and Diabetes, Department of Pediatrics and Adolescent Medicine, Ulm University Medical Center, 89075 Ulm, Germany; 5 Department of Biochemistry, Faculty of Pharmaceutical Sciences, Niigata University of Pharmacy and Medical and Life Sciences, Niigata, 956-8603 Japan; 6 Division of DNA Repair and Genome Integrity, Faculty of Medical Technology, Niigata University of Pharmacy and Medical and Life Sciences, Niigata, 956-8603 Japan; 7 RNA Therapeutics Division, Faculty of Medical Technology, Niigata University of Pharmacy and Medical and Life Sciences, Niigata, 956-8603 Japan; 8 Graduate School of Science, Kanagawa University, Yokohama, 221-8686 Japan; 9 Division of Anatomy and Histology, Faculty of Medical Technology, Niigata University of Pharmacy and Medical and Life Sciences, Niigata, 956-8603 Japan

**Keywords:** Adipogenesis, Polypeptide *N*-acetylgalactosaminyltransferase, *GALNT15*, Preadipocytes, Adipocytes, Developmental biology, Obesity

## Abstract

Adipogenesis involves intricate molecular mechanisms regulated by various transcription factors and signaling pathways. In this study, we aimed to identify factors specifically induced during adipogenesis in the human preadipocyte cell line, SGBS, but not in the mouse preadipocyte cell line, 3T3-L1. Microarray analysis revealed distinct gene expression profiles, with 1460 genes induced in SGBS cells and 1297 genes induced in 3T3-L1 cells during adipogenesis, with only 297 genes commonly induced. Among the genes uniquely induced in SGBS cells, we focused on *GALNT15*, which encodes polypeptide *N*-acetylgalactosaminyltransferase-15. Its expression increased transiently during adipogenesis in SGBS cells but remained low in 3T3-L1 cells. Overexpression of *GALNT15* increased mRNA levels of CCAAT-enhancer binding protein (C/EBPα) and leptin but had no significant impact on adipogenesis in SGBS cells. Conversely, knockdown of *GALNT15* suppressed mRNA expression of adipocyte marker genes, reduced lipid accumulation, and decreased the percentage of cells with oil droplets. The induction of C/EBPα and peroxisome proliferator-activated receptor γ during adipogenesis was promoted or suppressed in SGBS cells subjected to overexpression or knockdown of *GALNT1*5, respectively*.* These data suggest that polypeptide *N*-acetylgalactosaminyltransferase-15 is a novel regulatory molecule that enhances adipogenesis in SGBS cells.

## Introduction

Obesity is a condition characterized by the excessive accumulation of adipose tissue in the body, which, along with its common complications such as, type 2 diabetes, dyslipidemia, and hypertension, increases the risk of vascular diseases related to arteriosclerosis^1^. Adipose tissue comprises mature adipocytes with oil droplets that store triglycerides, and stromal vascular fractions containing preadipocytes, which are precursor cells of adipocytes. Excessive accumulation of adipose tissue is thought to result from the hypertrophy of individual adipocytes due to increased triglyceride accumulation, and an increased number of adipocytes due to the accelerated differentiation of preadipocytes into mature adipocytes^2^.

During adipogenesis, preadipocytes acquire the machinery for lipid transport and synthesis, insulin sensitivity, and secretion of adipocyte-specific proteins^2^. During this process, the induction and activation of transcription factors, specifically the CCAAT-enhancer binding protein (C/EBP) family and peroxisome proliferator-activated receptor γ (PPARγ), are crucial^2,3^. C/EBPβ and C/EBPδ are transiently induced early in adipogenesis^4^ and they induce expressions of C/EBPα and PPARγ, which function to promote transcription of many types of genes involved in adipocyte phenotype and function^2,3^. In addition to these key transcriptional regulators, various other transcriptional regulators of adipogenesis and factors affecting adipogenesis have been reported. While many of these findings regarding adipogenesis have been obtained using *in vitro* mouse models utilizing cell lines, such as 3T3-L1 or 3T3-F442A, the mechanisms of adipogenesis in humans and mice are not entirely identical. For instance, 3T3-L1 and 3T3-F442A cells undergo an increase in cell number known as mitotic clonal expansion prior to differentiation, which is thought to be necessary for subsequently differentiation. However, human primary preadipocytes or SGBS cells, a human preadipocyte cell line established from the subcutaneous adipose tissue of an infant suspected of having Simpson-Golabi-Behmel Syndrome^5^, do not necessarily undergo mitotic clonal expansion during adipogenesis^6,7^. Furthermore, LIM domain only 3 (*LMO3*), which positively regulates adipogenesis, is upregulated during adipogenesis in humans but not in mice^8^. Additionally, D-dopachrome tautomerase, an adipokine secreted by adipocytes, suppresses adipogenesis in SGBS cells but not in 3T3-L1 cells^9^. Thus, identifying the factors that affect adipogenesis in a human-specific manner may lead to the identification of novel target molecules for the development of anti-obesity drugs.

In the present study, we conducted a comparative analysis of mRNA expression induced during adipogenesis in SGBS cells and 3T3-L1 cells using microarray to identify genes whose expression is induced in SGBS but not in 3T3-L1 cells. *GALNT15*, encoding polypeptide *N*-acetylgalactosaminyltransferase (GalNAc-T)-15, was identified as one of the candidate genes. *GALNT15*, a member of the *GALNT* family comprising 20 species in humans, is expressed in most human tissues^10^. GalNAc-T15 catalyzes the initiation of mucin-type *O*-linked glycosylation by adding *N*-acetylgalactosamine to the serine or threonine residues of polypeptides^10^. Mucin-type *O*-glycosylation is initiated and regulated by the GalNAc-T family that catalyzes the first step in the biosynthesis forming the GalNAcα1-*O*-serine/threonine linkage in *O*-glycoproteins. *O*-linked glycosylation, the most diverse form of post-translational modifications, affects various aspects of protein function, therefore, many GalNAc-Ts are considered to have potentials for differential regulation in cells and tissues^11^. Aberrant *O*-glycosylation by some GalNAc-Ts has been observed in many types of cancer and is associated with noncancerous developmental and metabolic disorders^12,13^; however, the involvement of *GALNT15* in these diseases has not been reported, and its physiological function remains largely unknown. Therefore, we focused on *GALNT15* and investigated its effects on adipogenesis in SGBS cells.

## Results

### GALNT15 expression was induced during adipogenesis in SGBS but not in 3T3-L1 cells

**Fig. 1:**
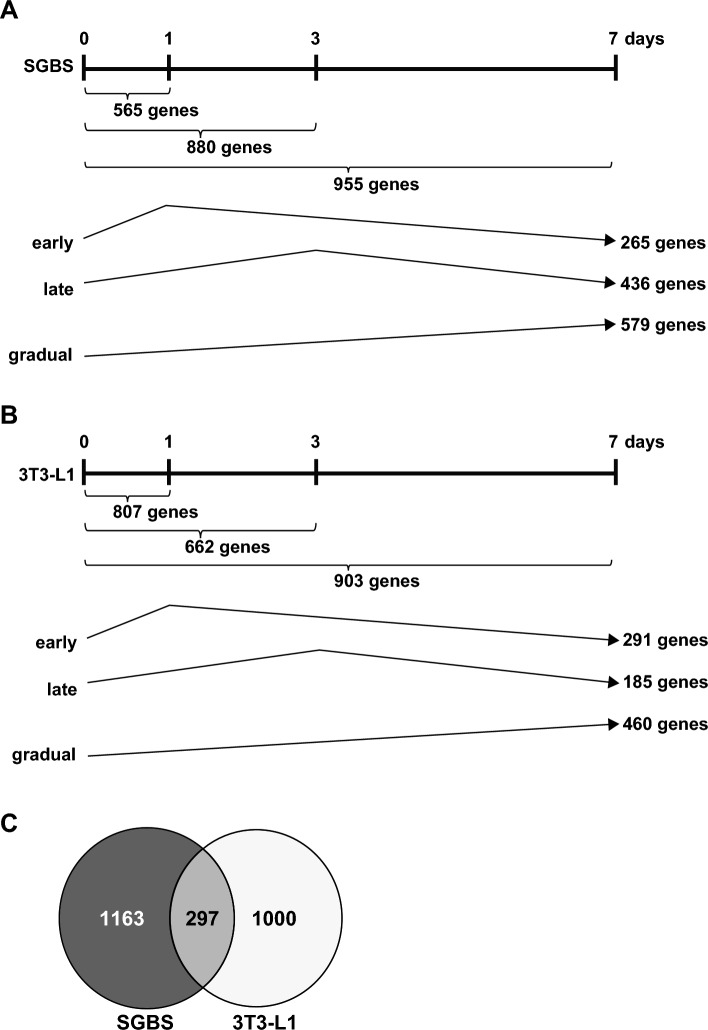
Identification of genes with increased expression during adipogenesis in SGBS and 3T3-L1 cells using microarray. The numbers represent the count of genes that increased more than 2-fold after adipogenic induction at day 1, 3, and 7, compared with that before induction (day 0) in SGBS cells (**A**) and in 3T3-L1 cells (**B**). Genes are classified into "early", "late", and "gradual" based on their expression induction patterns during adipogenesis, with the number of genes in each category also shown. (**C**) A Venn diagram illustrating the overlap between number of genes upregulated more than 2-fold after adipogenic induction at either day 1, 3, or 7 compared with that before induction in SGBS cells and in 3T3-L1 cells.

**Fig. 2:**
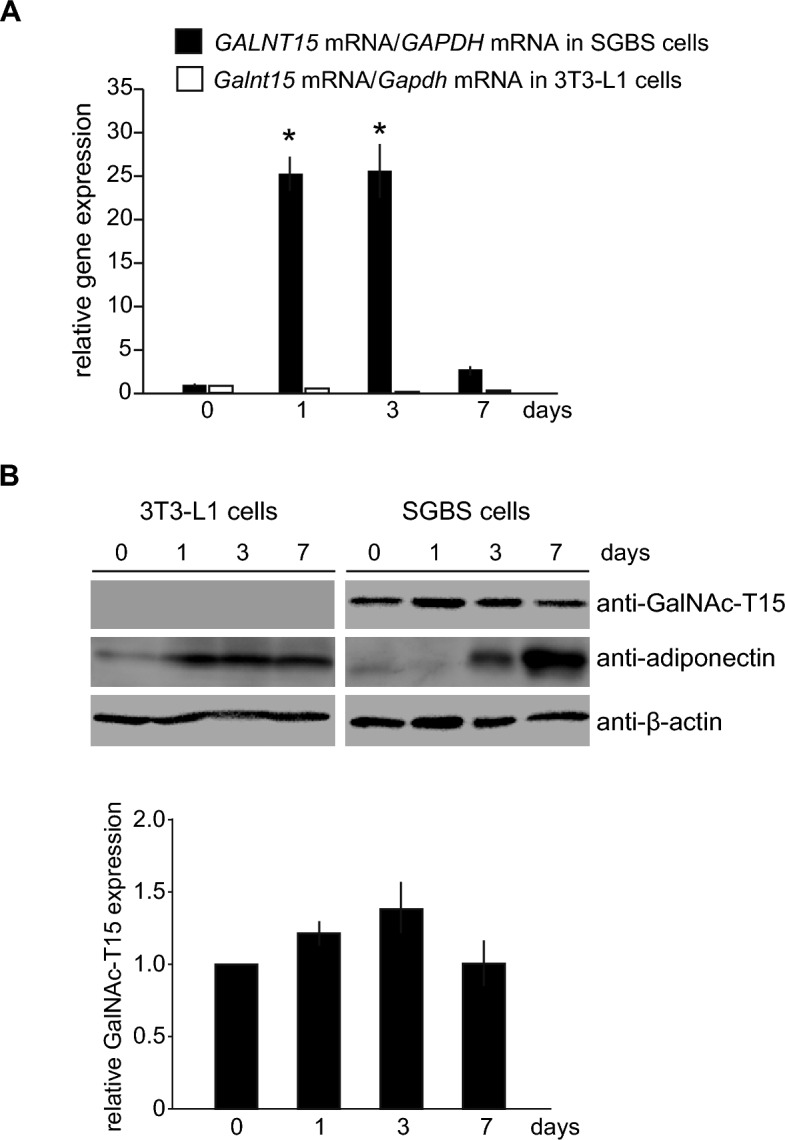
*GALNT15/Galnt15* expression during adipogenesis in SGBS and 3T3-L1 cells. (**A**) *GALNT15/Galnt15.* mRNA expression after adipogenic induction at 0, 1, 3, and 7 days in SGBS cells (black columns) and 3T3-L1 cells (white columns). Data represents relative values compared with those before adipogenesis in each cell type. *P < 0.05 (n = 3). (**B**) Representative western blotting images using lysates from SGBS and 3T3-L1 cells subjected to adipogenic induction for the indicated days, probed with an anti-GalNAc-T15 antibody. Anti-adiponectin antibody and anti-β-actin antibodies were used as positive controls for adipogenesis and internal controls, respectively. The graph illustrates the average band density of GalNAc-T15 normalized to β-actin in SGBS cells from four western blotting, with day 0 set to 1 as relative values. There were no statistically significant differences observed.

Within our experimental setup, the mRNA expression of fatty acid binding protein 4, an adipocyte marker, has been peaked around day 7 in both 3T3-L1 and SGBS cells after adipogenic induction (Supplementary Fig. S1). Therefore, the mRNA expression profiles of SGBS and 3T3-L1 cells on days 0, 1, 3, and 7 after adipogenic induction were analyzed using microarray analysis. Genes whose expression was upregulated by more than 2-fold compared with that in cells before adipogenic induction (day 0) were extracted. In SGBS cells, 565, 880, and 955 genes were upregulated on days 1, 3, and 7, respectively, after adipogenic induction (Fig. 1A). Excluding overlapping genes, 1460 genes were identified as induced during adipogenesis in SGBS cells. These genes were further classified as “early” (peak on day 1), “late” (peak on day 3), and "gradual" (expression continuing up to day 7), resulting in 436 “early”, 265 “late”, and 579 “gradual” genes identified as being induced during adipogenesis in SGBS cells. Similarly, in 3T3-L1 cells, 807, 662, and 903 genes were induced on days 1, 3, and 7 after adipogenic induction, respectively (Fig. 1B), resulting in 1297 induced genes during adipogenesis and classified as 185 “early”, 291 “late”, and 460 “gradual” genes. In both cell types, only 297 genes were found to be commonly induced (Fig. 1C), which included well-known adipocyte marker genes, such as *ADIPOQ* (adiponectin)*, CEBPA* (C/EBPα)*, DGAT2* (diacylglycerol *O*-acyltransferase 2)*, FABP4* (fatty acid binding protein 4)*,* and *PPARG* (PPARγ) (Table 1). Among the genes induced exclusively in SGBS cells, 61 exhibited more than 20-fold induction, including *LMO3,* whose expression is reported to be induced during adipogenesis in humans but not in mice^8^. Among these, we focused on *GALNT15* due to its relatively high expression at day 0, large variation in expression levels, and its poorly understood function in most organs, including adipose tissue. Furthermore, considering that other members of the *GALNT* family were not induced during adipogenesis in either SGBS cells or 3T3-L1 cells (Supplementary Table S1), we speculated that *GALNT15* may have a specific role in adipocyte differentiation. Microarray analysis revealed that the signal intensity of *GALNT15* in SGBS cells increased 63.73-, 37.05-, and 1.30-fold on days 1, 3, and 7 after adipogenic induction, respectively (Table 1). By contrast, the signal intensity of *Galnt15* in 3T3-L1 cells showed minor fluctuations, with fold changes of 1.02, −1.07, and 1.09, respectively, at the same time points following adipogenic induction. Quantitative reverse transcription polymerase chain reaction (qRT-PCR) validated these findings, highlighting a transient increase in *GALNT15* mRNA levels during adipogenesis in SGBS but not in 3T3-L1 cells (Fig. 2A). Western blotting also confirmed a similar pattern, with GalNAc-T15 protein levels transiently tending to increase in SGBS cells but barely detected throughout adipogenesis in 3T3-L1 cells (Fig. 2B).

**Table 1:** Fold changes and classification of microarray probeset signals for representative genes induced at day 1, 3, and 7 after adipogenic induction.

Gene symbol (human/mouse)	SGBS cells				3T3-L1 cells			
	Day 1	Day 3	Day 7	Class	Day 1	Day 3	Day 7	Class
*ADIPOQ/Adipoq*	−1.10	164.85	425.87	Gradual	1.09	27.14	299.76	Gradual
*AGT/Agt*	1.16	3.29	22.47	Gradual	13.49	17.38	35.45	Gradual
*CEBPA/Cebpa*	1.20	24.03	67.72	Gradual	1.56	1.81	3.43	Gradual
*CIDEC/Cidec*	4.16	48.59	111.01	Gradual	2.62	40.81	111.06	Gradual
*DGAT2/Dgat2*	1.22	8.23	23.62	Gradual	−1.27	3.36	7.59	Gradual
*FABP4/Fabp4*	3.11	637.63	1053.8	Gradual	54.42	407.06	597.63	Gradual
*GALNT15/Galnt15*	63.73	37.05	1.30	Early	1.02	−1.07	1.09	
*IGFBP4/Igfbp4*	1.25	32.68	11.95	Late	3.53	3.94	3.05	Late
*LIPE/Lipe*	1.08	3.28	14.74	Gradual	3.64	4.82	7.04	Gradual
*LMO3/Lmo3*	22.42	44.06	6.62	Late	1.06	1.10	-1.01	
*LPL/Lpl*	1.10	128.67	362.19	Gradual	1.31	6.27	8.18	Gradual
*PPARG/Pparg2*	5.84	17.43	19.45	Gradual	2.24	2.97	5.36	Gradual
*RGS2/Rgs2*	5.66	1.81	1.77	Early	11.19	12.2	8.09	Late

*GALNT15* is an "early" gene whose expression may be influenced by components present in the medium used for adipogenic induction in SGBS cells but absent in the medium used for adipogenic induction in 3T3-L1 cells. Therefore, we investigated which components of the medium used for adipogenic induction in SGBS cells affect *GALNT15* mRNA expression. The absence of fetal bovine serum (FBS) and glucocorticoids such as cortisol and dexamethasone (DEX) increased *GALNT15* mRNA expression (Supplementary Fig. S2).

### GALNT15 overexpression enhances CEBPA and LEP mRNA expression in SGBS cells

**Fig. 3:**
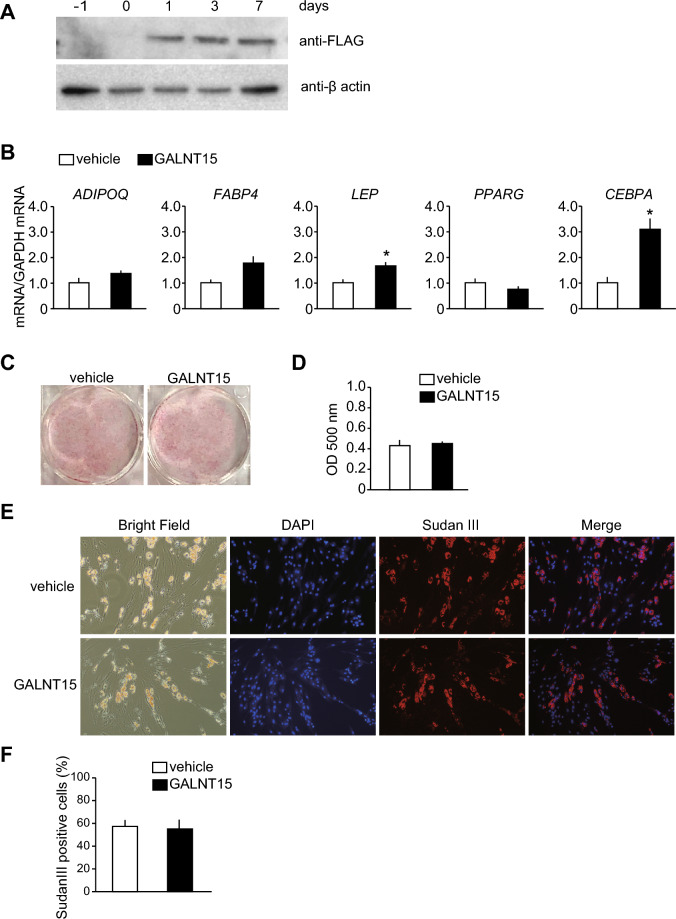
Effects of *GALNT15* overexpression on adipogenesis in SGBS cells. SGBS cells were infected with adenovirus overexpressing GalNAc-T15-FLAG fusion protein (*GALNT15*; black columns) or adenovirus without insertion (vehicle; white columns) for 24 h, followed by adipogenic induction. Adipogenesis was evaluated on day 7 (**B**) or day 10 (C-F) after adipogenic induction. (**A**) The expression of GalNAc-T15-FLAG fusion protein during adipogenesis in SGBS cells. Cell lysate from SGBS cells before the adenovirus infection (-1 day) and at the indicated day after adipogenic induction were probed with an anti-FLAG antibody using western blotting. An anti-β-actin antibody was used as an internal control. (**B**) mRNA expression of adipocyte marker genes in SGBS cells subjected to adipogenic induction. The graph represents relative values compared with those in control cells. *P < 0.05 (n = 3). (**C**, **D**) Lipid accumulation in SGBS cells subjected to adipogenic induction. Representative images (**C**) and the colorimetric quantification (**D**) in SGBS cells stained with Oil Red O are shown. N = 3. (**E**, **F**) Percentage of cells with oil droplets in SGBS cells subjected to adipogenic induction. Representative images (**E**) and graph of percentage (**F**) in SGBS cells stained both by 4,6-diamidine-2-phenylindole dihydrochloride (DAPI) and Sudan III are presented. The graph represents data averaged across three different fields (x100) of view.

To investigate the impact of *GALNT15* on adipogenesis in SGBS cells, we first constructed an adenovirus overexpressing the GalNAc-T15-FLAG fusion protein and assessed adipogenesis in SGBS cells transfected with this virus compared with that in cells transfected with a control virus (vehicle). Western blotting demonstrated the ectopic expression of the GalNAc-T15-FLAG fusion protein in SGBS cells (Fig. 3A). *GALNT15* overexpression significantly increased the mRNA expressions of *CEBPA* and *LEP*, but not those of *ADIPOQ, FABP4*, and *PPARG*, in SGBS cells 7 days after adipogenic induction (Fig. 3B). The amount of triglyceride accumulation and the percentage of cells with oil droplets were comparable between *GALNT15*-overexpressing and control cells (Fig. 3C, D).

### GALNT15 knockdown inhibits adipogenesis in SGBS cells

**Fig. 4:**
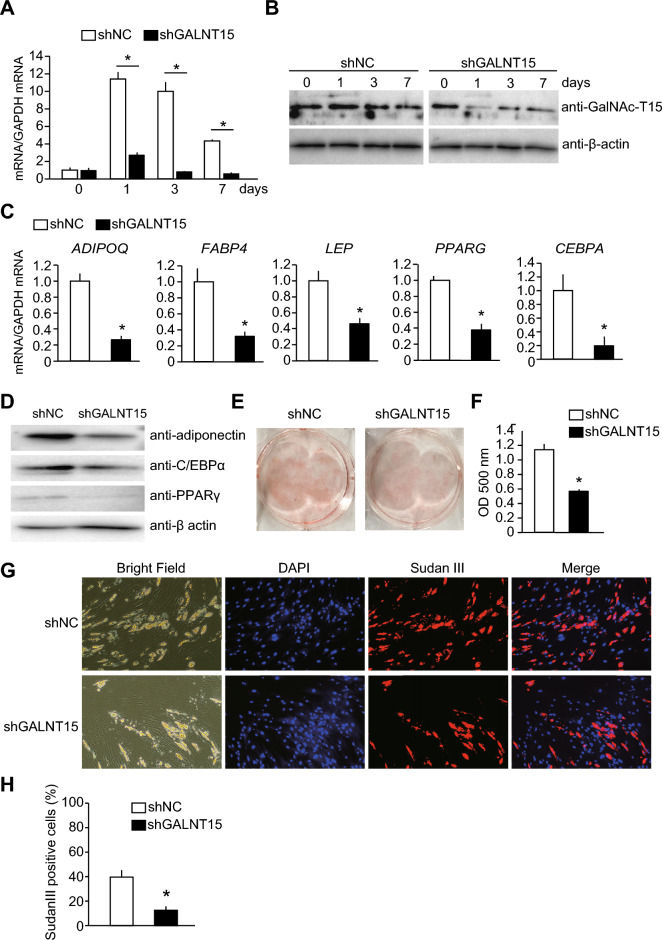
Effects of *GALNT15* knockdown on adipogenesis in SGBS cells. SGBS cells were infected with adenovirus expressing shRNA against *GALNT15* mRNA (shGALNT15; black columns) or control adenovirus expressing negative control shRNA (shNC; white columns) for 24 h, followed by adipogenic induction. Adipogenesis was evaluated on day 7 (**C**, **D**) or day 10 (E-H) after adipogenic induction. (**A**, **B**) Suppression of *GALNT15* mRNA (**A**) and GalNAc-T15 (**B**) expressions by shGALNT15 during adipogensis in SGBS cells was confirmed by quantitative reverse transcription polymerase chain reaction (**A**) and western blotting (3T3-L1 cells), respectively. The graph represents relative values compared with those before adipogenesis in control cells. *P < 0.05 (n = 3). (**C**) mRNA expression of adipocyte marker genes in SGBS cells subjected to adipogenic induction. The graph represents relative values compared with those in control cells. *P < 0.05 (n = 3). (**D**) Representative western blotting images using lysates from SGBS cells subjected to adipogenic induction, probed with an anti-adiponectin, anti-C/EBPα, and anti-PPARγ antibodies. Anti-β-actin antibody was used as an internal control. (**E**, **F**) The effect on lipid accumulation in SGBS cells subjected to adipogenic induction was assessed by Oil Red O staining. Representative images (**E**) and the colorimetric quantification (F) of SGBS cells stained by oil red O are shown. *P < 0.05 (n = 3). (**G**, **H**) The effect on the percentage of cells with oil droplets in SGBS cells subjected to adipogenic induction. Representative images (**G**) and the percentage (H) of SGBS cells stained with both 4,6-diamidine-2-phenylindole dihydrochloride (DAPI) and Sudan III are presented. *P < 0.05 (n = 3).

Subsequently, we constructed adenoviruses expressing a short hairpin RNA (shRNA) against *GALNT15* mRNA (shGALNT15) and a non-targeting control shRNA (shNC) and assessed adipogenesis in transfected SGBS cells. The induction of *GALNT15* mRNA and protein expressions during adipogenesis was inhibited in SGBS cells transfected with the adenovirus expressing shGALNT15 (Fig. 4A, B). *GALNT15* knockdown suppressed mRNA expression of all tested adipocyte marker genes (Fig. 4C), as well as the protein expression of adiponectin, C/EBPα, and PPARγ (Fig. 4D), triglyceride accumulation (Fig. 4E, F), and the percentage of cells with oil droplets (Fig. 4G, H), indicating that *GALNT15* knockdown inhibited adipogenesis in SGBS cells.

### GALNT15 enhances the induction of PPARG and CEBPA during adipogenesis

**Fig. 5:**
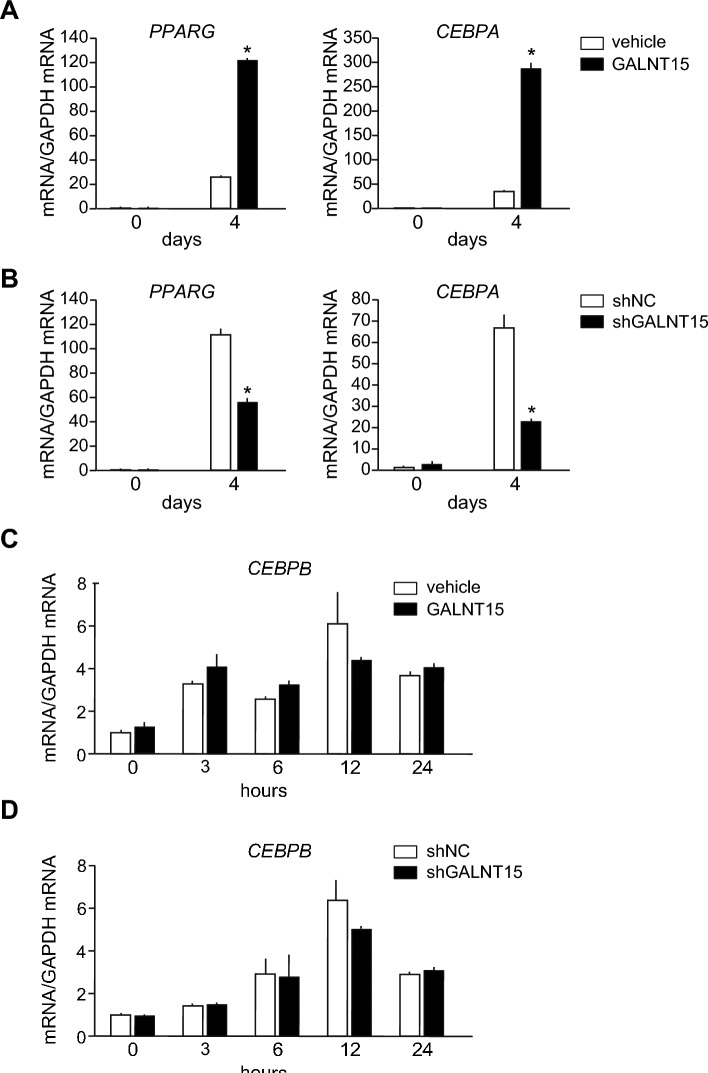
Effect of *GALNT15* overexpression or knockdown on the induction of *PPARG*, *CEBPA*, and CEBPB mRNA expression during SGBS adipogenesis. SGBS cells were infected with adenovirus overexpressing GalNAc-T15-FLAG fusion protein (*GALNT15*: black columns in (**A**) and (**C**), adenovirus expressing shGALNT15 (black columns in B and D), or corresponding control adenovirus (white columns) for 24 h, followed by adipogenic induction. mRNA expression was measured on day 4 for *PPARG* (**A**, **B**) and at 3, 6, 12, 24 h for *CEBPB* (**C**, **D**) after adipogenic induction and compared with that of cells before adipogenic induction. The data represents relative values compared with those before adipogenesis in each control cell. *P < 0.05 (n = 3).

To elucidate the molecular mechanisms by which *GALNT15* participates in adipogenesis, we examined the mRNA levels of two key adipogenic transcriptional regulatory genes, *PPARG* and *CEBPA*, during adipogenesis. *GALNT15* overexpression significantly enhanced the induction levels of both *PPARG* and *CEBPA* mRNA during adipogenesis in SGBS cells (Fig. 5A), whereas *GALNT15* knockdown suppressed the induction of *PPARG* and *CEBPA* mRNA 4 days after adipogenic induction compared with that in control cells (Fig. 5B). These results indicate that *GALNT15* is involved in adipogenesis by enhancing the expression of *PPARG* and *CEBPA* during adipogenesis in SGBS cells. Furthermore, the effect of *GALNT15* on the mRNA expressions of *CEBPB*, a transcription factor upstream of *PPARG* and *CEBPA*, in the early stages of adipogenesis was investigated. Neither overexpression nor knockdown of *GALNT15* altered the expression of *CEBPB* mRNA in SGBS cells at 0, 3, 6, 12 and 24 h after adipogenic induction (Fig. 5C, D), suggesting that mechanisms other than inducing the expression of *CEBPB* are involved in the regulation of *PPARG* and *CEBPA* by *GALNT15*.

## Discussion

Microarray analysis revealed distinct expression patterns of many genes during adipogenesis in human SGBS cells and mouse 3T3-L1 cells. This may be attributed to species differences between humans and mice; however, there may also be other differences in their properties as preadipocytes. For instance, the profile of genes expressed by differentiated adipocytes derived from 3T3-L1 cells is markedly different from that expressed by mature adipocytes in mouse^15^. By contrast, the mRNA expression profile of adipocytes derived from SGBS cells is similar to that of primary human white subcutaneous adipocytes^16–18^. Nevertheless, numerous molecules implicated in adipogenesis have been identified and validated using 3T3-L1 cells. Thus, identifying genes induced during the adipogenic process in SGBS cells, but not in 3T3-L1 cells in this study, could serve as a viable strategy for identifying adipogenic factors as yet undiscovered in humans. In fact, among the 61 genes exhibiting more than 20-fold induction during the SGBS adipogenic process, but not in 3T3-L1 cells, some genes, except for *GALNT15*, whose involvement in adipogenesis is unknown to our knowledge, were included. Notably, with *LMO3*, a human-specific adipogenic gene^8^, being one of these 61 genes, there is a possibility that unidentified adipogenic factors may also be present within this group.

In this study, *GALNT15* is identified as a gene induced during the adipogenesis of SGBS cells, consistent with reports indicating that *GALNT15* is listed as one of upregulated during adipocyte differentiation from human adipose-derived stem cells^19^. The induction of GalNAc-T15 protein during adipogenesis in SGBS cells was less pronounced compared to the induction of *GALNT15* mRNA, possibly due to post-transcriptional modifications, translation efficiency, mRNA stability, or other factors. However, the exact mechanisms are not extensively covered in this study. Although the mRNA expression of *Galnt15* was not induced, and protein expression was not detected during adipogenesis of 3T3-L1 cells, it cannot be ruled out that the induction was undetectable due to extremely low expression levels compared with those in SGBS cells. The regulation of *Galnt15* gene expression is influenced by corticosterone and the stress response in the mouse hippocampus^20^. There is a distinction in the presence of FBS between the media employed for adipogenic induction of SGBS cells and 3T3-L1 cells. While FBS deficiency increased *GALNT15* mRNA expression, probably due to cell stress, glucocorticoids appear to have a stronger impact on *GALNT15* expression. Despite the presence of DEX in the medium used for adipogenic induction in 3T3-L1 cells, no induction of *Galnt15* expression was observed during the adipogenic process, suggesting that the transcriptional regulation of *GALNT15/Galnt15* might differ between humans and mice. This study focused on the identification of novel human adipogenesis-related factors, therefore, the investigation in mice was limited to confirming *Galnt15* expression in 3T3-L1 cells. However, it is necessary to carefully consider whether a transient increase in the expression of *Galnt15* is observed during mouse adipogenesis or whether *Galnt15* is also involved in mouse adipogenesis.

Although inhibition of *GALNT15* induction clearly impeded adipogenesis in SGBS cells, overexpression of *GALNT15* did not affect the accumulation of triglycerides or the proportion of cells containing lipid droplets, despite affecting the induction of *PPARG* mRNA 4 days after adipogenic induction in SGBS cells. This suggests that the induced expression levels of *PPARG* during the adipogenic process in control SGBS cells may be sufficient to affect adipogenesis under our experimental conditions, with further overexpression potentially having no additional effect on these aspects. Furthermore, overexpression of *GALNT15* enhanced only the mRNA expression of *CEBPA* and its direct target gene *LEP*^21^, among the tested adipogenic marker genes. The role of *CEBPA* in adipogenesis is limited to the induction and maintenance of *PPARG* expression and the establishment of insulin sensitivity^22^. This suggests that the lack of adipogenic promotion in SGBS cells overexpressing *GALNT15* could be attribute to sufficient levels of *PPARG* expression. Conversely, *GALNT15* knockdown may affect adipogenesis by suppressing *CEBPA* expression, resulting in insufficient *PPARG* expression levels for adipogenic differentiation. Additionally, the high basal levels of GalNAc-T15 in SGBS cells may explain the absence of adipogenic promotion observed with *GALNT15* overexpression. Western blotting revealed the presence of GalNAc-T15 protein in SGBS cells prior to adipogenic induction, suggesting that the endogenous levels of GalNAc-T15 might be sufficient to support adipogenesis, even in the absence of exogenous *GALNT15* overexpression. On the other hand, shGALNT15 likely reduced both the basal and induced levels of GalNAc-T15 during adipogenesis, strongly providing clearer insights into the functional role of *GALNT15* in adipogenesis.

Abnormal *O*-GalNAc-glycosylation catalyzed by the *GALNT* family is associated with various human diseases, with particular attention focused on the link between *GALNT2* and metabolic disorders, such as obesity, type 2 diabetes, and lipid abnormalities^23^. *In vitro* analysis has shown that a reduction in *GALNT2* expression in HepG2 cells, a human hepatocarcinoma cell line, impairs insulin signaling and action^24^. Conversely, *GALNT2* overexpression stimulates adipocyte maturation and enlargement in 3T3-L1 cells^25^. However, our microarray data revealed that, except for *GALNT15*, other *GALNT* family members did not exhibit a remarkable increase during the adipogenic process in SGBS cells. This may suggest a more profound role for *GALNT15* than for *GALNT2* in human adipogenesis. *GALNT15* does not show a significant relation with other *GALNT* family member^11^, and to our knowledge, its physiological function has not been thoroughly investigated. Our findings, combined with the fact that *GALNT15* also serves as a marker gene candidate during osteocyte differentiation from canine adipose derived stem cells^26^, suggest that *GALNT15* may play an important role in the differentiation of mesenchymal stem cells.

In conclusion, we have demonstrated that *GALNT15* contributes to adipogenesis in SGBS cells by upregulating *CEBPA* and *PPARG*. However, the specific molecular mechanisms driving *GALNT15* -induced adipogenesis, including the potential involvement of unidentified substrates or non-enzymatic functions of GalNAc-T15, remain unclear and require further investigation for comprehensive elucidation. Our findings suggest that *GALNT15* is an attractive drug target for the treatment of obesity.

## Methods

### Cell culture and adipogenic induction

SGBS cells provided by our co-author, Dr. Martin Wabitsch, Ulm University Medical Center, Germany, were cultured in 6-well plates with culture medium consisting of Dulbecco’s modified Eagle’s medium/Ham’s F-12 medium (DMEM/F12; Fujifilm, Tokyo, Japan) supplemented with 10% FBS (Sigma, St Louis, MO, USA), 3 µM biotin, 17 µM pantothenic acid, and 0.5% penicillin-streptomycin-amphotericin B suspension (Fujifilm) in an incubator at 37 °C with humidified air at 5% CO_2_. To induce adipogenesis, cells grown to 80–90% confluency were cultured with FBS-free medium containing 0.01 mg/ml transferrin, 0.1 µM cortisol, 200 pM triiodothyronine, 20 nM insulin, 0.25 µM DEX, 500 µM 3-isobutyl-1-methylxanthine, and 2 µM troglitazone for 4 days. Subsequently, the cells were cultured in a maintenance medium consisting of FBS-free medium containing 0.01 mg/ml transferrin, 0.1 µM cortisol, 200 pM triiodothyronine, and 20 nM insulin. The maintenance medium was changed every 3 days.

3T3-L1 cells were generously provided by Oral Bioscience Laboratory, Tokushima University. Japan. The cells were cultured in 6-well plates with culture medium consisting of Dulbecco’s modified Eagle’s medium (Fujifilm) supplemented with 10% FBS and 0.5% penicillin-streptomycin-amphotericin B suspension. To induce adipogenesis, cells grown to 100% confluency were cultured for an additional 2 days and then cultured in medium containing 10 μM insulin, 1 µM DEX, 500 µM 3-isobutyl-1-methylxanthine, and 2 µM troglitazone for 3 days. Subsequently, the cells were cultured in maintenance medium consisting of medium containing 10 μM insulin. The maintenance medium was changed every 3 days.

### Microarray

Total RNA was extracted from SGBS and 3T3-L1 cells before (day 0) and on days 1, 3, and 7 after adipogenic induction, using ISOGEN (Nippongene, Toyama, Japan). The extracted RNA was used to generate biotin-labeled cRNA using the Affymetrix GeneChip^TM^ 3′ IVT PLUS Reagent Kit (Thermo Fisher Scientific, Waltham, MA, USA). The biotin-labeled RNA was then hybridized to either an Affymetrix Human Genome U-219 Array plate (Thermo Fisher Scientific) or a mouse genome MG-430 PM array plate (Thermo Fisher Scientific) following the manufacturer’s instructions. After washing and staining the array strips, the signals were developed and scanned using the Affymetrix Gene Atlas system (Thermo Fisher Scientific), and the data were analyzed using Transcriptome Analysis Console software (Thermo Fisher Scientific). Average hybridization signal intensities were used for data analysis, and genes with a mean signal intensity greater than 5 (log base 2 scale) in either of the adipogenic-induced samples were considered detectable. Genes with a signal intensity more than 2-fold higher than that in each cell before adipogenic induction were considered induced genes, and comparisons were made between SGBS and 3T3-L1 cells. Genes with the same symbol in humans and mice were designated as common genes.

### qRT-PCR

Each cDNA was synthesized from total RNA using the ReverTra Ace^®^ qPCR RT Kit (Toyobo, Osaka, Japan) following the manufacturer's protocol. The cDNA was then subjected to qRT-PCR on a Thermal Cycler Dice^®^ Real Time System (Takara, Shiga, Japan) using Thunderbird^TM^ SYBR^®^ qPCR Mix (Toyobo) and gene-specific primer sets via the following program: 30 sec at 95 °C, followed by 40 cycles of 95 °C for 15 sec and 60 °C for 1 min. The specificity of each primer set was confirmed by dissociation curve analysis following amplification. The nucleotide sequences of the primer sets are listed in Supplementary Table S2. The mRNA level of each gene was normalized to that of the human and mouse glyceraldehyde 3-phosphate dehydrogenase gene (*GAPDH/Gapdh*).

### Western blotting

The cells were lysed with lysis buffer (50 mM Tris (pH 7.4), 150 mM NaCl, 1 mM ethylenediamine tetraacetic acid, 1% Triton X-100, and complete mini (Roche, Basel, Switzerland)), and the lysates were subjected to sodium dodecyl sulfate-polyacrylamide gel electrophoresis and transferred to polyvinylidene fluoride membranes (Immobilon Transfer Membranes; Millipore, Bedford, MA, USA). After incubation in blocking solution (Blocking One; Nakalai tesque, Kyoto, Japan), the membranes were incubated with a 1:1000 dilution of mouse anti-FLAG M2 antibody (Sigma), a 1:2000 dilution of mouse anti-β actin (Sigma), a 1:500 dilution of rabbit anti-GalNAc-T15 antibody (Thermo Fisher), a 1:1000 dilution of mouse anti-adiponectin antibody (Proteintech, Rosemont, IL, USA), a 1:1000 dilution of rabbit anti-C/EBPα antibody (Proteintech), or a 1:500 dilution of mouse anti-PPARγ antibody (Proteintech), and subsequently incubated with an anti-rabbit or anti-mouse IgG-horseradish peroxidase-conjugated secondary antibody (Jackson Lab, Farmington, CT, USA). The signal was detected using Immobilon Western Detection Reagent (Millipore) with a Luminograph III (Atto, Tokyo, Japan). Band intensity was measured using ImageJ (ver.1.53t) program.

### Construction of adenoviruses

Adenoviruses expressing GalNAc-T15 fused to FLAG at the C-terminus and a shGALNT15 were constructed as previously described^14^. Briefly, cDNA encoding the translational region lacking the stop codon of *GALNT15* was amplified from SGBS adipocyte cDNA using PCR, and DNA with FLAG cDNA sequences added to its 3'-end was inserted into the pAxCAwtit cosmid vector (TakaRa). Recombinant adenoviral genomic DNA was excised from the cosmid and transfected into HEK293 cells to produce an adenovirus. An adenovirus produced from intact pAxCAwtit was used as a control. Cosmids for adenovirus production, which were inserted with a cDNA encoding shGALNT15 (target sequence: 5'-AGTCTGCTCTCAGCGAATATG-3', vector ID: VB900074-6932bqd) and a cDNA encoding a shNC (target sequence: 5'-CCTAAGGTTAAGTCGCCCTCG,-3' vector ID: VB010000-9479yzr), were purchased from Vector Builder (Chicago, IL, USA), and adenoviruses were produced in the same manner.

### Evaluation of adipogenesis

The degree of adipocyte differentiation was evaluated based on the expression of adipocyte marker genes, triglyceride accumulation, and the percentage of cells with oil droplets. SGBS cells were infected with each adenoviruses for 24 h and subjected to adipogenic induction. Seven days after adipogenic induction, total RNA was extracted from the cells and the expression of adipocyte marker genes was measured by qRT-PCR. Ten days after adipogenic induction, SGBS cells were fixed with 4% paraformaldehyde and stained with Oil Red O to evaluate triglyceride accumulation or 4,6-diamidine-2-phenylindole dihydrochloride and Sudan III to count cells with oil droplets. To measure the amount of triglycerides, stained Oil Red O was eluted with isopropanol, and the absorbance was measured at 500 nm using a spectrophotometer (Ultrospec 6300 pro; GE Healthcare, Chicago, IL, USA). To assess the percentage of cells with oil droplets, the ratio of Sudan III-positive cells to 4,6-diamidine-2-phenylindole dihydrochloride-stained cells was determined in 3 randomly selected low-power fields (x100).

### Data analysis

Each experiment in Figs. 2, 3, 4, 5 was repeated several times, and representative results are shown. Each bar on the graph is expressed as the mean ± SE. Statistical analyses were performed using Student’s *t*-test for the comparison of two groups and Dunnett's test for the comparison of three or more groups versus the control. Differences were considered significant when the *P* value was less than 0.05.

### Supplementary Information

Supplementary Figure S1.

Supplementary Figure S2.

Supplementary Information.

Supplementary Table S1.

Supplementary Table S2.

## Data Availability

The datasets generated during and/or analyzed during the current study are available from the corresponding author on reasonable request.
