# Supplementary material for: Polypeptide N-acetylgalactosaminyltransferase-15 regulates adipogenesis in human SGBS cells
Source: Sci Rep. 2024 Aug 29;14:20049. doi: 10.1038/s41598-024-70930-5 (PMC11362553; doi:10.1038/s41598-024-70930-5)
Supplement: Supplementary file 1 — Supplementary Figure S1. [file 41598_2024_70930_MOESM1_ESM.docx]

**Supplementary Fig. S1** Expression of FABP4/Fabp4 mRNA, an adipocyte marker, in SGBS cells (A) and 3T3-L1 cells (B) at the indicted days after adipogenic induction. Data represents relative values compared with those before adipogenesis in each cell type. Statistical analysis was performed using Dunnett's test for the comparison of each group versus the control (day 0). **P*<0.05 (n=3).
