# Supplementary material for: Polypeptide N-acetylgalactosaminyltransferase-15 regulates adipogenesis in human SGBS cells
Source: Sci Rep. 2024 Aug 29;14:20049. doi: 10.1038/s41598-024-70930-5 (PMC11362553; doi:10.1038/s41598-024-70930-5)
Supplement: Supplementary file 2 — Supplementary Figure S2. [file 41598_2024_70930_MOESM2_ESM.docx]

**Supplementary Fig. S2** The effects of FBS absence and individual components of adipogenic differentiation medium on GALNT15 mRNA in SGBS cells. GALNT15 mRNA levels were assessed in SGBS cells cultured in medium without FBS (FBS(-)) or in medium which included either 20 nM insulin (Ins), 200 pM triiodothyronine (T3), 0.1 µM cortisol (Cor), 0.25 µM dexamethasone (DEX), 500 µM 3-isobutyl-1-methylxanthine (IBMX), 2 µM troglitazone (Tro), or 0.01 mg/ml transferrin (TF) for 24 h by qRT-PCR. Data represents relative values compared with those in cells cultured with normal medium (FBS+). Statistical analysis was performed using Dunnett's test for the comparison of each group versus the control (FBS(+)). **P*<0.05 (n=3).
