# Supplementary material for: Polypeptide N-acetylgalactosaminyltransferase-15 regulates adipogenesis in human SGBS cells
Source: Sci Rep. 2024 Aug 29;14:20049. doi: 10.1038/s41598-024-70930-5 (PMC11362553; doi:10.1038/s41598-024-70930-5)
Supplement: Supplementary file 3 — Supplementary Information. [file 41598_2024_70930_MOESM3_ESM.docx]

**Supplementary Information**

The original Western blot images used for each figure, along with the approximate extent of the cropped area, are shown. PC denotes the positive control sample.
