# Supplementary material for: Polypeptide N-acetylgalactosaminyltransferase-15 regulates adipogenesis in human SGBS cells
Source: Sci Rep. 2024 Aug 29;14:20049. doi: 10.1038/s41598-024-70930-5 (PMC11362553; doi:10.1038/s41598-024-70930-5)
Supplement: Supplementary file 4 — Supplementary Table S1. [file 41598_2024_70930_MOESM4_ESM.docx]

| Gene symbol  (human/mouse) | SGBS cells | | | 3T3 L1 cells | | |
| --- | --- | --- | --- | --- | --- | --- |
|  | day 1 | day 3 | day 7 | day 1 | day 3 | day 7 |
| *GALNT1/Galnt1* | 1.08 | -1.02 | -1.01 | 1.69 | 1.46 | 1.53 |
| *GALNT2/Galnt2* | 1.03 | -1.29 | 1.4 | 1.30 | 1.28 | 1.25 |
| *GALNT3/Galnt3* | -1.07 | 1.26 | -1.03 | 1.19 | 1.18 | 1.33 |
| *GALNT4/Galnt4* | 1.48 | 1.04 | 1.09 | 1.51 | 1.18 | 1.55 |
| *GALNT5/Galnt5* | -1.76 | -1.91 | -2.14 | 1.02 | 1.03 | 1.11 |
| *GALNT6/Galnt6* | -1.29 | -1.05 | -1.34 | 1.12 | 1.13 | 1.03 |
| *GALNT7/Galnt7* | 1.07 | -2.61 | -2.27 | -2.02 | -1.64 | -1.98 |
| *GALNT8/Galnt8* | 1.18 | 1.16 | 1.09 | 1.31 | 1.18 | 1.3 |
| *GALNT9/Galnt9* | 1.2 | 1.24 | 1.24 | 1.15 | 1.19 | 1.14 |
| *GALNT10/Galnt10* | -2.21 | -3.08 | -2.31 | 1.34 | 1.23 | 1.44 |
| *GALNT11/Galnt11* | 1.2 | 1.53 | 1.52 | 1.18 | 1.11 | 1.10 |
| *GALNT12/Galnt12* | -1.98 | -1.77 | -3.22 | 1.15 | 1.15 | -1.05 |
| *GALNT13/Galnt13* | 1.2 | 1.11 | 1.27 | 1.10 | 1.05 | 1.09 |
| *GALNT14/Galnt14* | -1.08 | -1.01 | -1.01 | -1.09 | -1.12 | -1.19 |
| *GALNT16/Galnt16* | 1.08 | 1.44 | 1.05 | 1.03 | 1.09 | 1.01 |
| *GALNT17(WBSCR17)/*  *Galnt17(Gcap8)* | 1.17 | 1.1 | 1.07 | -1.16 | -1.13 | 1.16 |
| *GALNT18/Galnt18* | 1.01 | -1.02 | -1.08 | -1.2 | -1.56 | -1.27 |
| *GALNT19(GALNTL5)/*  *Galnt19(Galntl5)* | -1.05 | 1.3 | 1.08 | 1.15 | 1.19 | 1.14 |
| *GALNT20(GALNTL6)/*  *Galnt20(Galntl6)* | 1.08 | 1.34 | 1.29 | 1.19 | 1.14 | -1 |

**Supplementary Table 1**  Fold changes of the microarray probeset signals for *GALNT* family genes on days 1, 3, and 7 after adipogenic induction in SGBS cells and in 3T3-L1 cells.
