# Supplementary material for: Polypeptide N-acetylgalactosaminyltransferase-15 regulates adipogenesis in human SGBS cells
Source: Sci Rep. 2024 Aug 29;14:20049. doi: 10.1038/s41598-024-70930-5 (PMC11362553; doi:10.1038/s41598-024-70930-5)
Supplement: Supplementary file 5 — Supplementary Table S2. [file 41598_2024_70930_MOESM5_ESM.docx]

| **Gene** | **forward primer** | **reverse primer** |
| --- | --- | --- |
| human *ADIPOQ* | 5'-cctaagggagacatcggtga-3' | 5'-gtaaagcgaatgggcatgtt-3' |
| human *CEBPA* | 5'-aaccttgtgccttggaaatg-3' | 5'-ccctatgtttccaccccttt-3' |
| human *CEBPB* | 5'-agctgctccaccttcttctg-3' | 5'-gacaagcacagcgacgagta-3' |
| human *FABP4* | 5'-tactgggccaggaatttgac-3' | 5'-gtggaagtgacgcctttcat-3' |
| human *GAPDH* | 5'-gaaggtgaaggtcggagtc-3' | 5'-gaagatggtgatgggatttc-3' |
| human *GALNT15* | 5'-gcaaatgtcaggaccaggtt-3' | 5'-gcaggaggaactggagtctg-3' |
| human *LEP* | 5'-gaagaccacatccacacacg-3' | 5'-agctcagccagacccatcta-3' |
| mouse *Galnt15* | 5'-tggattggaagctggatttc-3' | 5'-gatcgtatgctccggtgttt-3' |
| mouse *Gapdh* | 5'-aactttggcattgtggaagg-3' | 5'-acacattgggggtaggaaca-3' |

**Supplementary table 2** Nucleotide sequences of primer sets used for qRT-PCR.
